# Breast cancer screening in sub-Saharan Africa: a systematic review and ethical appraisal

**DOI:** 10.1186/s12885-022-09299-5

**Published:** 2022-02-23

**Authors:** Yehoda M. Martei, Bege Dauda, Verna Vanderpuye

**Affiliations:** 1 Department of Medicine (Division of Hematology-Oncology), University of Pennsylvania, Philadelphia, PA USA; 2 Botswana UPenn Partnership, Gaborone, Botswana; 3 Center for Global Genomics and Health Equity, University of Pennsylvania, Philadelphia, PA USA; 4 National Center for Radiotherapy Oncology and Nuclear Medicine, Korle-Bu Teaching Hospital, Accra, Ghana

## Abstract

**Background:**

The aim of this systematic review was to evaluate the evidence and clinical outcomes of screening interventions and implementation trials in sub-Saharan Africa (SSA) and also appraise some ethical issues related to screening in the region through quantitative and qualitative narrative synthesis of the literature.

**Methods:**

We searched Pubmed, OvidMEDLINE, Embase, and Web of Science to identify studies published on breast cancer screening interventions and outcomes in SSA. Descriptive statistics were used to summarize the frequency and proportions of extracted variables, and narrative syntheses was used to evaluate the clinical outcomes of the different screening modalities. The mixed methods appraisal tool was used to assess the quality of studies included in the review.

**Results:**

Fifteen studies were included, which consisted of 72,572 women in ten countries in SSA. 63% (8/15) of the included publications evaluated Clinical Breast Examination (CBE), 47% (7/15) evaluated mammography and 7% (1/15) evaluated ultrasound screening. The cancer detection rate was < 1/1000 to 3.3/1000 and 3.3/100 to 56/1000 for CBE and mammography screening respectively. There was a lot of heterogeneity in CBE methods, target age for screening and no clear documentation of screening interval. Cost-effective analyses showed that CBE screening linked to comprehensive cancer care is most cost effective. There was limited discussion of the ethics of screening, including the possible harms of screening in the absence of linkage to care. The gap between conducting good screening program and the appropriate follow-up with diagnosis and treatment remains one of the major challenges of screening in SSA.

**Discussion:**

There is insufficient real-world data to support the systematic implementation of national breast cancer screening in SSA. Further research is needed to answer important questions about screening, and national and international partnerships are needed to ensure that appropriate diagnostic and treatment modalities are available to patients who screen positive.

**Supplementary Information:**

The online version contains supplementary material available at 10.1186/s12885-022-09299-5.

## Background

Breast cancer is the most common cause of cancer death among women globally with a disproportionate burden of mortality in developing countries [1]. Whereas breast cancer survival rates are increasing in most developed countries, in part due to early detection and more effective treatment [2–4], Sub-Saharan Africa (SSA) has the worst mortality-to-incidence ratios globally [2]. This has been partly attributed to advanced stage at presentation with approximately 80% of patients presenting with locally advanced and metastatic disease at diagnosis [5].

Efforts to reduce the burden of disease in SSA has focused on improving survival by increasing the rates of early detection for breast cancer, combined with effective treatment for early-stage disease in order to improve the cure rates and survival for breast cancer. In developed countries mammography screening annually or biennially has significantly reduced breast cancer mortality rates by at least 20% [6], and is accepted as a gold standard for cost effective breast cancer screening. The World Health Organization (WHO) currently recommends systematic mammography screening for women between ages 40 to 75 years through population-based mammography screening program in well-resourced settings [7, 8]. However, in resource-limited settings mammography has been assessed as not cost-effective and it is recommended that early detection focus on downstaging through improved breast cancer awareness [9]. Current research does not show a survival benefit of Breast Self-Examination (BSE) [10, 11] and Clinical Breast Examination (CBE) [12], but may be promising in resource-limited settings through downstaging [13], and if adequate diagnostic and therapeutic facilities are in place [8].

Currently, we are not aware of countries in SSA with a systematic national breast cancer screening program. The debate on the most appropriate screening modality for breast cancer in SSA has centered around the need to pursue global justice, which proposes equalizing access and utilization of mammography screening in less developed countries such as SSA [14], versus pursuing screening modalities that is informed by the cost-effective intervention trials and research performed within the socioeconomic context and the resources available within the current healthcare infrastructure [15]. Furthermore, there are ethical concerns about implementing screening interventions in SSA where there is limited or lack of access to diagnostic services, treatment, follow-up and an adequate number of healthcare professionals to accommodate the increased patient workload anticipated from screening. Given these challenges in resource-restricted settings, experts suggest that a greater priority now, is to provide interventions that are plausible in achieving early detection and adequate treatment for the cancers currently diagnosed [16].

The aim of this systematic review was to evaluate the evidence and clinical outcomes of screening interventions and implementation trials in SSA and also appraise some ethical issues related to screening in the region through quantitative and qualitative narrative synthesis of the literature. Findings from this study will contribute to the knowledge of effective screening interventions in SSA, and guide health policy and funding decisions on screening modalities appropriate in SSA.

## Methods

### Search strategy

The systematic review followed the recommendations of the Preferred Reporting Items for Systematic Reviews and Meta-Analysis (PRISMA) guidelines. An electronic search in Pubmed, OvidMEDLINE, Embase, and Web of Science was performed using Boolean search terms for *Clinical Breast Examination or Self Breast Examination* and *Africa* or mammogram* screen* and *Africa* or *breast cancer screening* and *Africa* or breast cancer screen* Africa. Studies were limited to research involving “Humans” and publications in English. There was no restriction on the year of publication and the search was carried out on articles up to April 24, 2019. Additional references were identified by reviewing the citation of key references. Publications that evaluated a screening intervention and clinical outcomes in asymptomatic women were included. Research conducted in countries outside of SSA were excluded, as well as publications that did not focus on breast cancer or included only symptomatic women. We also excluded publications that did not evaluate any of the following screening outcomes: survival, cancer cases diagnosed, stage shifting, positive predictive value and cost-effectiveness analysis (CEA). Reviews and conference proceedings were excluded. A two-step process was used to identify reviewed articles: Authors YMM and BD reviewed titles and abstracts that met inclusion criteria. Duplicates were removed and the full text articles were reviewed for data abstraction. All disagreements were resolved by further examination and discussions among the co-authors.

### Outcomes variables

The following variables were extracted: 1. Context (author, year, country); 2. Screening protocol (study group, interval,, longest follow-up); 3. Participants (*n* for study group and control (if applicable), age at enrollment); 4. Screening outcomes assessed and results. Ethical issues were qualitatively appraised, which included data on access to appropriate diagnostic and treatment for those who screen positive and assessment of harm-to-benefit ratio.

### Statistical analysis

Descriptive statistics were used to summarize the frequency and proportions of the extracted variables, and narrative synthesis was used to evaluate the clinical outcomes data of the included publications. The mixed methods appraisal tool (MMAT) [17] was used to assessed the quality of the studies included in the analysis. The assessment of quantitative non-randomized study was used to evaluate the quality of the included studies using five criteria.

## Results

### Study characteristics

**Fig. 1:**
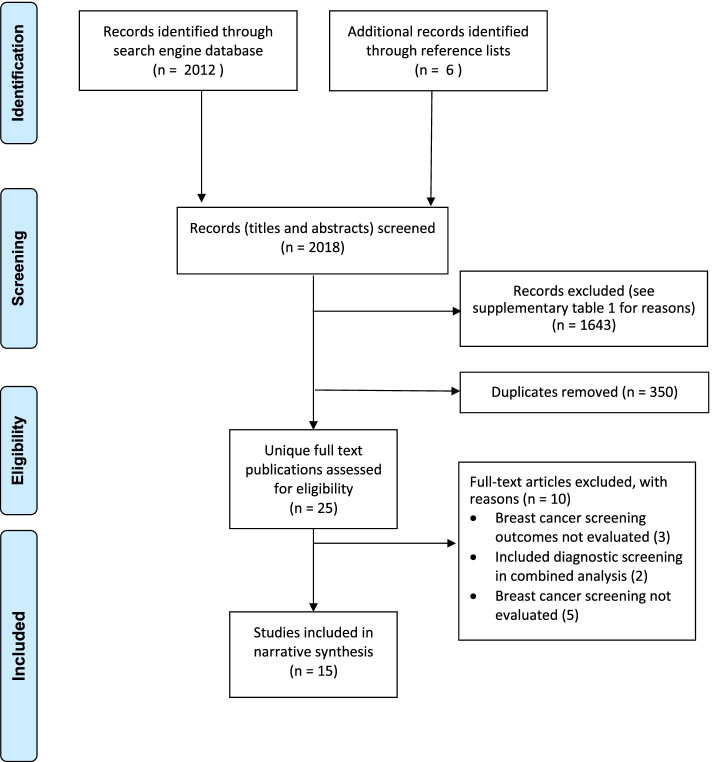
Flow chart of the study selection process

The flow chart for publications identified and reviewed for our analysis are presented in Fig. 1. 72,572 total participants (including 28,465 participants in the control group) were included from 15 articles, that evaluated screening intervention through research conducted in 10 countries and published from year 2008 to year 2018. This included three publications on CEA using mathematical modeling and one publication on cancer outcomes assessment using microsimulation. 63% (8/15) of the included publications evaluated CBE, 47% (7/15) evaluated mammography and 7% (1/15) evaluated ultrasound screening. The screening outcomes assessed included cancer detection rates, stage shifting, CEA and survival outcomes. Two intervention studies included had a control arm, and one study was a cross-sectional self-controlled design. Details on the study population, screening modality and outcomes are presented in Table 1.

**Table 1:** Summary table with variables from included studies

Author (year), country	Screening protocol, interval, longest follow-up	Participants, *n* study group ; control group	Age at enrollment (years)	Relevant screening outcomes metrics measured and findings
Apffelstaedt [18] (2008), RSA	**Mammography**, retrospective review of a prospective cohort of opportunistic mammography screening *Interval:* NR *Longest follow-up:* NR	7638	Age ≥ 40	*Cancer cases diagnosed:* 7.2/1000 examinations
Abuidris [19] (2013), Sudan	**CBE,** conducted by trained local volunteers chosen from the community, using a cluster randomized design *Interval:* NR *Longest follow-up:* 34 months	14,788; 24,550	Age ≥ 18	*Cancer cases diagnosed:* 9 malignant + 8 DCIS (per 10,309 in the study group) *vs* 3 + 0 respectively control group; ~ < 1/1000 examinations
Brakohiapa [20] (2013), Ghana	**Mammography,** retrospective review of opportunistic mammography screening *Interval:* NR *Longest follow-up:* 1 year	106	NR	*Cancer cases diagnosed:* 6 IDC and 0 DCIS in the mammography screening group; ~ 56/1000 examinations
Luyeye Mvila [21] (2014), DRC	**CBE**, conducted by trained healthcare professionals, as part of a breast awareness campaign *Interval:* NR *Longest follow-up:* 34 months	4315 (CBE); 1113 (mammography)^a^	Age ≥ 18	*Cancer cases diagnosed:* 87 malignant + 13 DICS; *Stage shifting:* 24% Stage I & II; 75% stage III
Apffelstaedt [22] (2014), RSA	**Mammography**, utilizing opportunistic screening in a mobile breast screening unit *Interval:* NR *Longest follow-up:* 18 months	2712	Age ≥ 40	*Cancer cases diagnosed:* 3.71/1000 examinations
Apffelstaedt [23] (2014), RSA	**Mammography**, retrospective review of a prospective cohort of opportunistic mammography screening *Interval:* NR *Longest follow-up:* NR	3774	Age ≥ 40	*Cancer cases diagnosed:* 11.4/1000 examinations
Ngoma [24] (2015), Tanzania	**CBE**, conducted by trained lay personnel in a cluster randomized design *Interval:* annually *Longest follow-up:* 3 years	Y1 6686; 3915^b^ Y2 6534; 3915 Y3 6241; 3915	NR	*Stage Shifting:* Stage I&II in year 1,2 & 3 in the study group vs control group = 0,%, 33%, 50% (*p* = NS) vs 9%, 60%, 67% (*p* = 0.021) *Total expenditure:* $45,000 / year
Gutnik [25] (2016), Malawi	**CBE**, conducted by trained laywomen as breast health workers (BHW), in the clinic setting *Screening interval:* NR *Longest follow-up:* NR	1000	Age > 30	Cancer cases diagnosed*: 2 per 1000 examinations *PPV of CBE exam:* 48% for BHW compared to physician exam
Sayed [26] (2016), Kenya	**CBE**, conducted by healthcare professionals in “breast awareness camps in the hospital setting *Screening interval:* NR *Longest follow-up:* NR	833	Age ≥ 15	*Cancer cases diagnosed:* 2 per 1000 exams in asymptomatic women
Omidiji [27] (2017), Nigeria	**Mammogram *vs* ultrasound,** conducted by radiologists. self-controlled cross-sectional design *Screening interval:* NR *Longest follow-up:* NR	300	Age 30—60	*Cancer cases diagnosed:* 1 IDC and 6 DCIS per 300 asymptomatic women; 3.3 per 1000 examinations *Positive predictive value:* 33.3% for ultrasound compared with mammogram
Pinder [28] (2018), Zambia	**CBE**, conducted by trained healthcare professionals *Interval:* NR *Longest follow-up:* NR	1955	NR	*Cancer cases diagnosed:* < 1% diagnosed with invasive cancer (17/1955); ~ 8.7 / 1000 examinations
Ginsberg [29] (2012), SSA	**Mammography,** cost effectiveness of mammography screening in a mathematically modelling study using WHO-CHOICE methods *Interval:* biennial *Longest follow-up:* NA	^c^	50—70	*CEA*: Biennial screening mammography + treatment of all stages cost between $Int2248 and $Int4596 per DALY averted
Zelle [30] (2012), Ghana	**CBE**, conducted by trained community nurses and screening **mammography** in a mathematical modelling study using WHO-CHOICE methods *Interval:* Biennial *Longest follow-up:* NA	^d^	40–69 (CBE) 50–69; 40–69 (Mammography)	*CEA:* Biennial CBE in women aged 40–69 + treatment for all stages seems the most cost effective—$1299 per DALY averted
Ralaidovy (2018), Eastern SSA [31]	**Mammography,** using “Generalized Cost-Effectiveness Analysis” *Interval:* Biennial *Longest follow-up:* NA	^e^	50–69	*ICER:* Biennial mammography in women 50–69 linked with timely diagnosis and treatment at 95% coverage – I$485 per HLY gained
Birnbaum [32] (2018), E. Africa (Uganda)	**CBE**, combined with multiple treatment strategies to assess breast cancer outcomes *Interval:* Annual *Longest follow-up:* 10-year cumulative outcomes	^f^	30 – 49 50—69	ARR: 113 (per 100,000 women) YLS: 418 (per 1000,000 women)

Key: *BHW* breast health workers, *NR* not reported, *PPV* positive predictive value, *CEA* cost effectiveness analysis, *IDC* invasive ductal carcinoma, *DCIS* ductal carcinoma in situ, *DALY* disability-adjusted life years, *HLY* healthy life years, *ARR* absolute risk reduction, *YLS* years life saved

^a^Respective proportions of screening vs diagnostic mammograms not reported

^b^Year 1 represents the baseline population the study and control villages

^c^Regional age-adjusted population estimates of breast cancer incidence, breast cancer prevalence. Percentage of prevalent cases treated, and background mortality rates were based on WHO Burden of Disease study estimates for 2000

^d^Population of female based on global burden of disease 2004 update

^e^Incidence estimates obtained from GLOBOCAN 2012

^f^International Agency for Research on Cancer. C15 I-X: Raikai, Uganda (2003–07)

### Quality of the studies

**Fig. 2:**
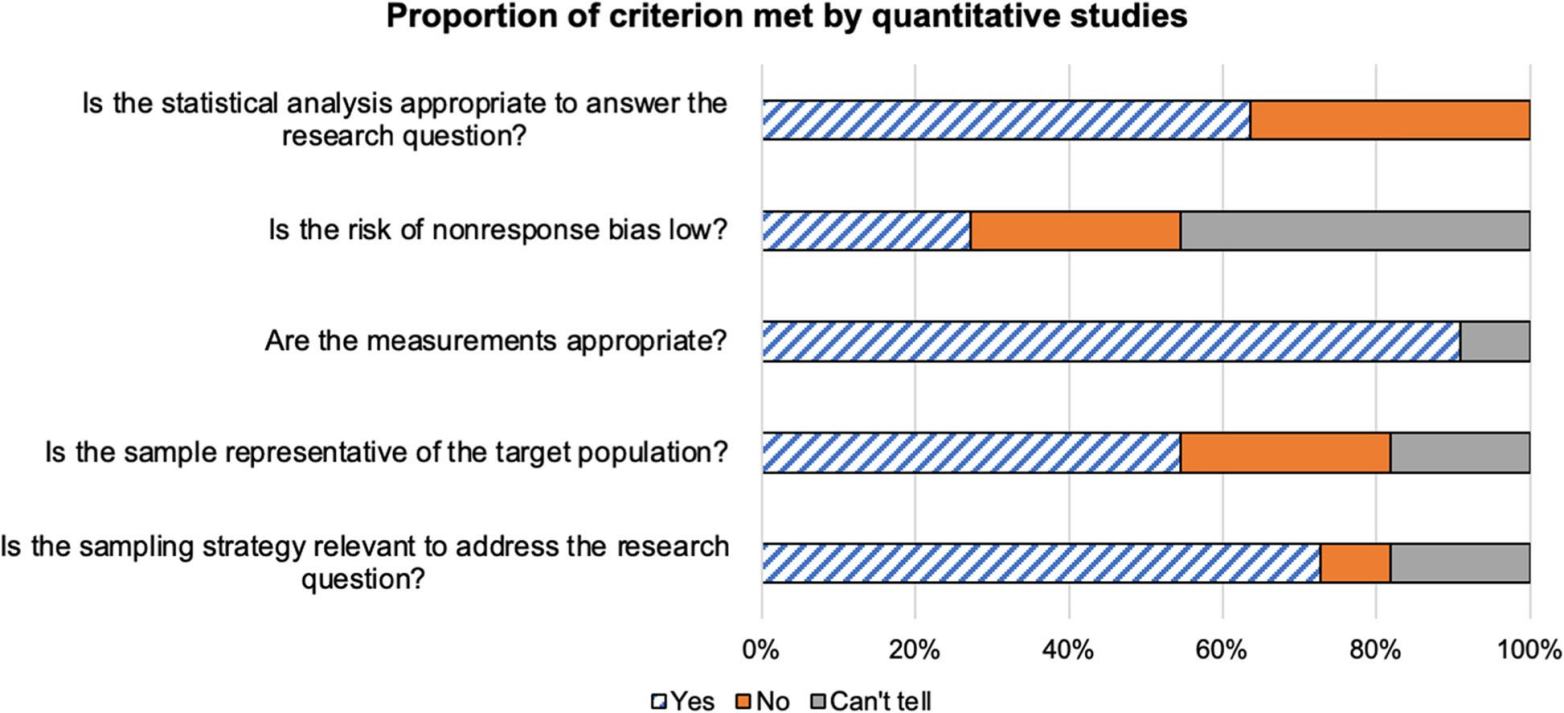
Distribution of quality criterion met by quantitative studies

The quality of the 11 quantitative studies included in our analysis was moderate as shown in Fig. 2. 88% of the publications used a sampling strategy relevant to the address the research question. Furthermore 67% of the articles used a sample representative of the target population. Three studies did not clearly define the age inclusion criteria for the participants enrolled in the study. Additionally, three studies evaluating breast cancer screening strategies included a lower limit of 15 years and 18 years for participants enrolled in the study. 33% of the publications had adequate documentation of risk of non-response, which was assessed as low. Finally, the majority of publications included in the review had adequate outcome data (> 80%) that were included in our analysis.

### Narrative synthesis

#### Clinical breast examination

There are few studies evaluating real-world data on clinical outcomes of breast cancer screening interventions in SSA. Majority of the studies were pilot studies and did not evaluate a control group in the study design. Of the studies included majority evaluate CBE, however there was significant heterogeneity in the personnel conducting these examinations – which included training of sampled lay persons, community healthcare workers (CHW) within the community that participants were selected from, and training of healthcare professionals. The setting also differed in terms of whether these were conducted in the community vs. the hospital or clinic setting or “breast camps.” The screening uptake rate differed across studies, with high screening uptake rate reported in studies that included extensive education and engagement of community leaders, religious groups, village leaders and in studies where the communities chose the candidates to be trained as CHW performing these examinations [21, 25, 33]. Interestingly, in Sudan, villages screened by volunteers from other villages had low screening uptake rate [33]. There was also disparate data in studies that reported higher uptake when CHW were financially compensated vs. not. The CBE studies were mostly pilots and did not evaluate different screening intervals or have long follow-up. In Tanzania, CBE uptake rates increased with subsequent years of annual screening evaluated [34].

The age of the target population at enrollment differed significantly for studies that reported this in their inclusion criteria. Of note studies have shown that more than half of patients diagnosed in SSA are ≤ 50 years [35]. Appropriately studies extended their inclusion criteria of women enrolled in asymptomatic screening. The lower limit at inclusion ranged from 15–30 years old.

There was also heterogeneity in the screening outcomes that were assessed and reported. The cancer diagnoses rate varied from < 1/1000 examinations to 8.7/1000 screened for CBE. This was unadjusted for the age groups of the women that were evaluated in each study. One showed a significantly higher proportion of early-stage disease (ie. stage I & II) in the study group compared to the control group [34]. One microsimulation study evaluated survival and absolute risk reduction in Eastern SSA. One study included total cost of the screening intervention only, which was assessed as $45,000 / year [34].

#### Mammography screening and ultrasound

Five studies included an assessment of mammography or ultrasound screening. These studies all evaluated opportunistic screening analyzed prospectively or retrospectively and one cross-sectional study. The studies varied in the age distribution of the population screened and the reported cancer detection rate that ranged from 3.3/1000 to 56/1000 examinations. None of these studies reported screening intervals assessed.

#### Cost effectiveness analysis

Three studies were modeling studies that used imputed data to analyze cost effectiveness of screening and treatment modalities for breast cancer in Ghana, Uganda (E. Africa region) and SSA. Although the methodology differed, the summarized data showed that the most cost-effective screening modality in SSA is biennial CBE and treatment of all stages for women aged 40–69, which corresponds to the highest reduction in disability-adjusted life years (DALY) and mortality. Mammography may be cost effective in parts of SSA that are assessed as middle-income countries and cost between $Int2248 and $4596 per DALY averted [29].

### Ethical appraisal

There were several ethical issues appraised from the studies included in our analysis. The data presented on breast screening in SSA remains insufficient to recommend systematic national screening programs in SSA. Further studies, specifically real-world data are needed to assess the clinical benefit of screening in this population. The benefit of screening can be realized in countries where there are integrated treatment programs established for breast cancer cases diagnosed early through screening [19]. Furthermore the benefit of downstaging and early detection can be fully achieved in programs where diagnostic and health system delays are minimal. In this regard, a model of breast cancer camps that included multidisciplinary teams that served as a one-stop shop for diagnosis may be most effective in SSA [26], given that several studies have shown prolonged systems delays for cancer patients [36–38]. Finally, there is no established age criteria for when CBE or mammography should be initiated in SSA, given that most patients present before age 50 [35]. This is important to assess including the psychological and associated costs of screening a lower limit of younger women whose risk of breast cancer does not make this a cost-effective intervention in that population. In studies that reported positive predictive value of the different modalities, a PPV for CBE by a trained laywoman compared to a physician had a 48% PPV, which raises the additional issue of the high rates false positive examinations and associated psychological implications. One study evaluated ultrasound screening, which is not an evidence-based screening modality and also reported PPV of 33%. All the studies evaluated expounded on the potential benefits of screening, but included limited discussion of potential harms.

## Discussion

This review showed that there are few articles that have examined breast cancer screening outcomes in SSA and the current data remains insufficient to recommend systematic national screening programs in any country in SSA. There is also a deficit of data analyzing the ethical issues of the effective rollout of national screening programs in SSA. Given the current debate surrounding breast cancer screening in developed countries, it is critical that future studies and recommendations that propose implementation of any breast cancer screening modality in SSA carefully evaluate the existing data for different screening strategies to ensure that the benefit to harm ratio is acceptable and cost-effective for the target populations being screened in order to realize significant survival benefits.

The following issues remain to be addressed by current screening interventions:

1. What is the best screening modality? The current data support that based on financial, human personnel and health infrastructure resources, CBE in the immediate future may be the most appropriate screening intervention in SSA. WHO does not recommend population-based mammography screening in limited-resource settings but offers consideration for CBE for women ages 50–69 in this setting [8]. An analysis of national cancer control plans globally, showed that only 5% and 18% of low-income countries and lower-middle-income plans respectively, included any population-based breast cancer screening [39]. Furthermore, few low- and middle-income counties have specific guidelines for CBE screening. For instance, Malaysia recommends CBE for women ≥ 35 years old and risk-stratified mammography screening programs starting at 40 years [40].
2. What target populations should be considered for screening? Majority of guidelines for mammography screening in developed countries recommend screening asymptomatic women aged ≥ 50 years. Screening recommendations from few low- and middle- income countries indicate that a lower age at screening initiation may be appropriate and generalizable to the SSA region where typically ≥ 50% of women present at age ≤ 50 years [35]. Screening interventions will therefore have to incorporate the at-risk population younger than 50, but assess a lower limit that does not include young women in whom the risk of breast cancer is low. In balancing this risk it is important to consider the prevention paradox, by assessing the specific risk distribution based on a country’s population distribution and breast cancer incidence and mortality pattern. Although there are no national population-based screening programs in SSA, the Cancer Association of South Africa recommends annual mammography screening starting at age 40 and biennial in women ≥ 55 years old [41]. There also some consideration for initiation of screening at 35 years such as current CBE guidelines in Malaysia [40], and based on recent data from a cluster randomized trial of CBE every two years in Indian women aged 35–64 years who were followed up for 20 years [13]. In a sub-analysis, women < 50 years who attended all prescribed rounds of screening had a breast cancer specific mortality benefit compared to no benefit in women who did not attend all rounds of screening [42].
3. What strategies are effective in increasing screening uptake? The data presented here shows that screening conducted by lay healthcare workers selected from the screening villages result in high screening uptake. Furthermore, CBE coupled with extensive mass campaigns and engagement of community/village leaders are also very effective. This is in line with the ethical recommendation of community engagement in resource poor settings. Community engagement serves as way of empowering the community to be active participants of intervention programs and ensuring that interaction with community is a continuous process even after the screening intervention is over [43]. Similarly, modelling breast cancer camps where multidisciplinary teams served as a one-stop shop for diagnosis underscores capacity building which is a vital requirement emphasized in resource poor settings. Collective approach to diagnosis and treatment encourages communication and exchange of ideas among team members which fosters knowledge, skills and expertise.

Subsequent studies need to provide clear data on training protocols for examiners, compensation, examiner-to-participant ratio, factors related to effective CBE uptake. Follow-up should be reported on linkage to diagnostic services. Finally, larger studies are needed to report significant differences in early-stage vs late-stage cancer diagnosis rate in the screened vs unscreened population, as well as survival and absolute risk reduction through screening. CEA should also incorporate more real-world data for the specific countries using actual healthcare costs within the public systems where available.

The main ethical challenge in screening interventions in SSA is the gap between conducting good screening program and the appropriate follow up with diagnosis and treatment. This challenge is similar to the one encountered in clinical trials in developing countries where availability of proven interventions is often not feasible or occur on a fragmented scale [44]. It is important therefore to develop research and advocate for policies that would incorporate screening and appropriate diagnosis and treatment. Achieving this will rely on fidelity to frameworks for ethical implementation of screening such as the Australia population-based screening framework [45], that are aligned with the WHO Screening Programmes guide, which advocates for a detailed contextual assessment of ethical considerations, benefits and potential harm prior to implementation of national systematic screening programs [46]. Most screening recommendations in SSA follow guidelines from North America and Europe, there is a critical need for a contextual adaption, which will ensure the incorporation of context specific values and true benefits of screening in improving the health outcomes of women. All studies should also address the potential harms of screening before recommending national screening programs. In our review, only two studies reported a positive predictive value of CBE. However, a complete assessment of harm should be incorporated into the initial study design and should include sensitivity and specificity evaluations. In SSA, elevated false positive rates may result in psychological distress and healthcare system strain due to multiple diagnostic follow-ups. Conversely high false negative rates from a suboptimally implemented or ineffective screening program may result in more prolonged diagnostic delays in a region where this is a significant problem [47]. In addition, diagnosing women at early stages can result in potential harm if there are delayed referrals and lack of access to adequate treatment which can be attributed to weak healthcare infrastructure in LMICs. Whereas the argument for overdiagnosis has remained central in the mammography screening debate [48], CBE is unlikely to increase diagnosis of in-situ only disease and therefore may be a lower consideration of the harm assessment in SSA. The variability of harm in different contexts further supports the need for contextual assessment in formulating screening policies in SSA. The question is less of whether there is a potential benefit and more of whether prior studies have addressed ethical considerations for implementing CBE screening in SSA. In fact, CEA analysis suggests that treatment alone of all stages in SSA might not be as cost effective, and that screening coupled with treatment of all stages constitute the most effective strategies. Further research and funding is needed to answer some of these important questions related to screening, and national government and international partnerships are needed to ensure that appropriate diagnostic and treatment modalities are available for breast cancer patients that are being diagnosed in their countries.

## Supplementary Information

**Additional file 1: Supplementary Table 1.** Reasons for exclusion of titles and abstracts from initial screen.

## Data Availability

All data generated or analysed during this study are included in this published article [and its supplementary information files].

## Abbreviations

ARR: Absolute Risk Reduction
BHW: Breast Health Worker
CBE: Clinical Breast Examination
CEA: Cost Effectiveness Analysis
CHW: Community Health Worker
DALY: Disability-Adjusted Life Years
DCIS: Ductal Carcinoma in situ
HLY: Healthy Life Years
IDC: Invasive Ductal Carcinoma
NR: Not Reported
PPV: Positive Predictive Value
SSA: Sub Saharan Africa
WHO: World Health Organization
YLS: Years Life Saved
