# Correction: Breast cancer screening in sub-Saharan Africa: a systematic review and ethical appraisal

**DOI:** 10.1186/s12885-022-09412-8

**Published:** 2022-03-22

**Authors:** Yehoda M. Martei, Bege Dauda, Verna Vanderpuye

**Affiliations:** 1 Department of Medicine (Division of Hematology-Oncology), University of Pennsylvania, Philadelphia, PA USA; 2 Botswana UPenn Partnership, Gaborone, Botswana; 3 Center for Global Genomics and Health Equity, University of Pennsylvania, Philadelphia, PA USA; 4 National Center for Radiotherapy Oncology and Nuclear Medicine, Korle-Bu Teaching Hospital, Accra, Ghana

**Correction to: BMC Cancer 22, 203 (2022)**

**https://doi.org/10.1186/s12885-022-09299-5**

Following publication of the original article [1], an error was identified in the Funding section. The updated funding statement is given below.

Dr. Yehoda Martei’s research work is supported by an NIH K01 Award—K01TW011481. Bege Dauda’s research is supported by the Penn Center for Global Genomics and Health Equity (PennGGHE) with funding from Genentech, Inc. and the Penn Medicine Office of Inclusion, Diversity, and Equity.

The original article [1] has been corrected.

## References

[1] Martei YM, Dauda B, Vanderpuye V (2022). Breast cancer screening in sub-Saharan Africa: a systematic review and ethical appraisal. BMC Cancer.

