# Supplementary material for: Breast cancer screening in sub-Saharan Africa: a systematic review and ethical appraisal
Source: BMC Cancer. 2022 Feb 23;22:203. doi: 10.1186/s12885-022-09299-5 (PMC8867875; doi:10.1186/s12885-022-09299-5)
Supplement: Supplementary file 1 — Additional file 1: Supplementary Table 1. Reasons for exclusion of titles and abstracts from initial screen. [file 12885_2022_9299_MOESM1_ESM.docx]

Supplementary Data

Supplementary Table 1: Reasons for exclusion of titles and abstracts from initial screen

| Reason for Exclusion | Number of Articles |
| --- | --- |
| Outside the scope of this article (eg. breast cancer genetics screening and molecular studies) | 936 |
| Knowledge, attitudes and practice / behavior focus | 189 |
| Barriers and facilitators of breast cancer presentation | 26 |
| Outside of Sub-Saharan Africa (including North Africa) | 149 |
| Breast cancer Case review / Epidemiology / Outcomes | 173 |
| Reviews / Commentaries / Consensus Guidelines | 125 |
| Gynecological cancers | 45 |
| **TOTAL** | **1643** |
